# Homœopathy in Military Hospitals

**Published:** 1862-02

**Authors:** 


### Selections.

### HOMŒOPATHY IN MILITARY HOSPITALS.

**Reprinted from *Am. Medical Times.***

The U. S. Senate is engaged, in these momentous times, in the consideration of a subject, in itself, perhaps, the most frivolous which ever enlisted the thoughts of a rational creature, but which may prove the most important act of the session. Senator Grimes, of Iowa, has introduced a bill placing some of the military hospitals, at Washington, under the charge of homœopathists. We do not know why this class of medical practitioners are honored with such distinction, and we think other systems have a just cause of complaint in being overlooked by a Government which they equally support, and which all are anxious to serve. If Government is about to institute experiments in its military hospitals, with a view to test medical theories, it does not appear why it should pass by Botanics, Hydropaths, Eclectics, Mesmerists, Kneisopaths, etc. Viewing the homœopathic system of practice from a rational, scientific stand-point, it must be regarded as the least worthy of attention of any now popular in this country. Indeed, we know of no system so indefensible as that which is engaging the attention of the honorable Senators. With no desire, however, to prejudge a question of so much importance, but earnestly seeking the welfare of sick soldiers, we deem it our duty to contribute to our legislators such information as may be in our possession, in the hope of aiding them in the formation of correct opinions as to the merits of the medical régime which they are urged to establish in our military hospitals.

This is not the first time that a government has been petitioned to recognize homœopathy, and grant it special privileges. Many European States have not only been thus petitioned, but have granted the prayers of the petitioners, and thoroughly tested its merits. The results of these trials will appear in the course of this article. Similar efforts to have public hospitals placed under their charge have also been made in this country by the partisans of Hahnemannism. On the occasion which we shall now notice, the whole subject was so thoroughly sifted, and the false pretensions of this system so completely exposed, that a quietus was put to their aspirations.

In the year 1857, a resolution was introduced into the Board of Governors of the Almshouse Department, New York, providing "that one-half of Bellevue Hospital should be set apart for the practice of homœopathy." A select committee was appointed to report upon the subject, of which the Hon. Washington Smith, one of the most intelligent civilians of the city, was chairman. The able Report, which this gentleman produced, bears evidence, on every page, of an unprejudiced review of the merits of the system, when thoroughly tested in hospital practice. We earnestly commend to the serious consideration of our HONORABLE SENATORS the following extracts from this report.

Alluding to the alleged claims of homœopathy on the ground of its popularity, the committee advance the following just opinions:

"That this system is wide-spread, and that it has adherents among the intelligent portions of the community, is an argument that applies with equal force to every system of medical empiricism. The opinion of a man of simply general intelligence, has properly no weight in regard to any new theory and its application to practice in any department of the arts or sciences. We should naturally look for a reliable opinion of the merits of such theory to the scientific cultivators of the art in which its application is proposed. Thus tested, the homœopathic system must utterly fail to receive our sanction. We appeal in vain to its adherents to point to a single medical man among its advocates in this city, whose scientific attainments in his own profession would entitle his opinion to our confidence. In no department of science is there more activity in the investigation of the principles upon which it is based, more acuteness in observation, or better logic in the deduction of practical precepts from such principles and observations, than in medicine. And yet the records of science show that all those who truly advance the several departments of medicine, all, without exception, both in this and foreign countries, belong to the ranks of the so-called regular system."

But the Board was urged to grant the request because so many petitions were presented to them from respectable citizens. To this suggestion the Report replies:

"But whence do these petitions emanate? Do they come to us from the inmates of the hospital who are to be the subjects of the experiment? Do the sick who crowd the wards complain of the incompetency of the medical officers, and of the inefficiency of their treatment, and petition us to change their medical attendants, and introduce a new system of practice? Do these petitions even emanate from the honest laboring classes of our city, whom the vicissitudes of life and the misfortunes of poverty may at any moment remove to the wards of Bellevue for relief from their bodily ills? These are questions which the Board would do well to ponder before it acts."

The body of the Report consists of a careful collation of evidence bearing on the propriety of introducing this system of practice into public hospitals.

"But we are not left to simple conjectures as to the actual success of homœopathy as a system of medical practice. It is our duty, however, to inquire simply as to its success in hospitals; and on this head statistics are sufficiently numerous to prove its entire inefficiency and utter failure wherever it has been tried. The following statistics have been collected with care from authentic sources:—

"In 1829, by order of the King of Naples, a commission was appointed to test homœpathic remedies, under the following restrictions:—1. The Commission shall consist of two professors of the University of the Faculty of Medicine, two members of the Medico-Chirurgical Academy, two members of public instruction, and the heads of the hospital. 2. The Commission, after having proved the attenuation of the homœpathic remedies, shall place the said remedies in a strong box, firmly closed, with two different locks, the keys of which shall be returned, one to the Director of the Clinique, and the other to the commissioners charged with following the treatment. 3. The clinical ward shall have but a single door, guarded by a sentinel; its internal arrangements shall be adapted to health; it shall not contain more than fifteen to twenty beds, and two assistant physicians, one chosen by the attending physician, the other by the commissioners, who shall keep an exact register of all that happens to the patients, the changes in their diseases, their regimen, cures and deaths, if any die. 4. The admission of patients affected with acute or chronic diseases, shall be left to the choice of the attending physician and commissioners, with this condition, that the attending physician shall not be obliged to take patients known to be incurable; nor shall diseases equivocal be considered proper for positive experiments. 5. The commissioners having selected the class of diseases, the attending physician shall make known the symptoms, administer the remedies, and prescribe the regimen. 6. Each day the condition of each patient shall be determined by the attending physician and commission. The result of this trial of forty days of homœopathic treatment under the observation of the commission named by the King of Naples, was the conclusion that not only is this treatment of no effect, but that in certain diseases it has the inconvenience of preventing the employment of remedies capable of effecting a cure. The physician in attendance was M. de Horatus, author of a homœopathic work, and who had boasted of the most marvellous cures.

"Clot-Bey, Physician-in-Chief to the armies of the Viceroy of Egypt states (*Annal. de la Med. Physiolog.*, Sept. 1834, *Ency.*, Decr. 1834) that a German homœopathic physician petitioned the Council of Health to try this system in the Hospital of Cairo, alleging its cheapness, etc. He was allowed to select, and chose patients suffering from opthalmia and dysentery.—The Council were convinced from this experiment that the Homœopathic system was not entitled to their confidence. The following is the conclusion of the Report of the Council of Health: That the cures obtained were due simply to the hygienic and dietetic treatment adopted, and not at all to the infinitesimal doses. So unsuccessful did this trial prove, that the homœopathic practitioner was obliged to abandon the country.

"In April, 1843, a ward with thirty beds in the Hotel Dieu de Lyon was placed in charge of M. Guerard, the most distinguished homæopathic physician of that city, with liberty to select his patients. He selected fifteen, suffering from febrile affections, pneumonias, erysipelas, catarrhs, etc. He visited them daily, and in presence of sixty students and several physicians, examined, prescribed homœopathic remedies, and directed the regimen. The experiment continued seventeen days, when the physician voluntarily retired. During this time there was no improvement in the patients, nor advantage gained which could be ascribed to the homæopathic treatment. The physician attributed his failure to the action of deleterious miasma *always existing in hospitals*, and from which he could not protect his patients. He acknowledged that the remedies which produced such powerful effects in private practice, utterly failed in hospitals, owing to the emanations from the bodies of persons collected together, which neutralized the infinitesimal doses.—*Gaz. Med. de Paris*, *Ency*. Nov. 1833.

"In 1834, M. Andral employed homœopathic remedies in one hundred and forty cases, in the Hospital de la Pitie of Paris. The arrangements of the ward, the regimen of the patients, and all the details of treatment, were carefully managed according to the directions of Hahnemann. The remedies were all obtained from the most eminent homœopathic apothecary in Paris, and administered with the most religious exactness. The result of this trial proved the entire inefficiency of the remedies employed. It was found necessary in most of the cases to resort finally to the regular treatment.—*Bull. Gen. de Therapeut.* 1834.

"In 1835, the Homœopathic Society of Paris petitioned the authorities to establish a Homœopathic Hospital and Dispensary. The minister referred the matter to the Academy of Medicine, which appointed a Commission to draw up a report. This Commission reported in substance as follows: That they had submitted the system of homœopathy to the most rigid tests in practice, without obtaining any other than negative results, so far as the action of remedies was concerned; while observation proved that great dangers were liable to follow its adoption in severe diseases, from the neglect of proper and reliable remedies. If the authorities yielded to this request, the advocates of Mesmerism, animal magnetism, etc., were equally entitled to have hospitals opened for the trial of their peculiar systems, and thus every form of quackery would demand attention. They therefore advised that the petition be not granted. The Minister of Public Instruction, acting upon the advice of this Report, refused the petition.

"In 1829, the Czar of Russia ordered that the system of homœopathy should be tried in several military hospitals. For several years the practice was continued, and reports of marvellous success were annually published, but it has entirely failed of obtaining the confidence of Government, and by a recent edict it is forbidden to practice homœopathy in the Russian territories.

"Homœopathy and allopathy were tried (*Ency. Jan.* 1836) in the Hospital of Fultschin for two months, with the following result:

| | Entered. | Cured. | Died. | Remaining. |
|---|---|---|---|---|
| In Allopathic Hospital, | 457 | 364 | — | 93 |
| " Homœopathic " | 129 | 66 | 5 | 58 |

"Piorry states that he has tried numerous experiments with homœopathic remedies in Hotel Dieu, all of which failed."—*Ency. Apr.* 1835, *Soc. Sav.* p. 88.

"Bally used homœopathic remedies four months in l'Hotel Dieu, with the following result:—pas un malade n'a été guéri par l'homœopathie."

"Dr. Guillott, of the Salpetriére, gave six beds to the homœopathists, in 1849, for the treatment of cholera. Of seven cases treated, all died.—*Lancet*, 1849, v. 4, p. 542.

"The per-centage of mortality in the Homœopathic Hospital of St. Petersburgh, 1833–34, was sixteen and two-thirds per cent.—*Ency. March*, 1835. *Rev. Med.* p. 41.

"Although homœopathy has existed nearly half a century and boasts of having overspread the civilized world, and received the special patronage of the wealthy of every community as well as a government sanction, it claims for itself to-day but seven hospitals in which it is practised on the entire continent of Europe; and within the last year or two several of these have been closed. The great Homœopathic Hospital of Vienna, which has published annually the most wonderful results of treatment, and as far as its reports gave evidence, was entirely successful, has recently ceased to exist.

"The Homœopathic Hospital at Leipsic, the home of the founder of this system, ceased with the death of Hahnemann. The London Homœopathic Hospital has recently closed its doors.

"But we need not multiply facts of this kind: enough has been given to prove to the entire satisfaction of your Committee, that this system has been thoroughly tested in hospitals, and found entirely inefficient. It is quite true that hospitals established by its partisans have published reports of the most flattering success of treatment, but they must be rejected in this discussion, because partisan. If such reports are reliable, why the failure of these very hospitals? Why is the homœopathic system expelled, not only from the hospitals of Russia, in which it has had years to establish itself, but even from the Czar's dominions?

These are questions of grave import, and may well give rise to the inquiry in this community, Why are the sick poor of our city, selected to be made the subjects of an experiment with this system of medical practice which has so repeatedly failed when put to test of rigid investigation? If the curiosity of the few *must* be gratified, why not choose the criminal for the experiment?"

With such facts before them, the Committee came to the following conclusion:

"The just pride of every civilized and Christian community is its public charities. They are not only the criterion by which we may estimate its Christian philanthrophy, but also its progress in the arts of civilized life. Well may the citizens of London, of Paris, and other continental cities boast of their hospitals, the growth of centuries, and the merited recipients of public and private endowments. To them flock the students of every country, and from them emanate men learned in the laws of health and disease, and skilled in all the subtle arts of healing. They are demonstrating with mathematical exactness the fact, that wisely and judiciously managed, the average of human life may be materially lengthened. So important, indeed, have they become to the well-being of the people, that they are incorporated with state and city governments. Well may we, under whose fostering care the public charities of our city are placed, inquire what is the character of the medical officers under which these hospitals have attained such celebrity! The answer, without exception is, that they are of the same school of education and practice as that under the management of which Bellevue Hospital has for the last ten years so signally prospered. They have been men of professional learning, eminent as citizens, and often as statesmen, but always of one school—the so-called regular practice."

No unprejudiced mind can review such facts, without concluding that public authorities who deliberately consign the helpless and confiding sick to the charge of medical men, practising a system so inefficient, incur a fearful responsiblity. And that responsibility assumes a tenfold importance when the sick, who are to be subjected to this experiment, are the citizen soldiers who have sacrificed the comforts of home in defence of their country. Around them Government should throw its protecting care, and tenderly guard their sick beds from the ruthless hand of medical charlatanism.

